# Can invertebrates see the e-vector of polarization as a separate modality of light?

**DOI:** 10.1242/jeb.139899

**Published:** 2016-12-15

**Authors:** Thomas Labhart

**Affiliations:** Institute of Molecular Life Sciences, University of Zurich, Winterthurerstrasse 190, Zürich CH 8057, Switzerland

**Keywords:** Invertebrates, Polarization vision, E-vector perception, Celestial compass, Polarization imaging, Polarization-opponent neurons

## Abstract

The visual world is rich in linearly polarized light stimuli, which are hidden from the human eye. But many invertebrate species make use of polarized light as a source of valuable visual information. However, exploiting light polarization does not necessarily imply that the electric (e)-vector orientation of polarized light can be perceived as a separate modality of light. In this Review, I address the question of whether invertebrates can detect specific e-vector orientations in a manner similar to that of humans perceiving spectral stimuli as specific hues. To analyze e-vector orientation, the signals of at least three polarization-sensitive sensors (analyzer channels) with different e-vector tuning axes must be compared. The object-based, imaging polarization vision systems of cephalopods and crustaceans, as well as the water-surface detectors of flying backswimmers, use just two analyzer channels. Although this excludes the perception of specific e-vector orientations, a two-channel system does provide a coarse, categoric analysis of polarized light stimuli, comparable to the limited color sense of dichromatic, ‘color-blind’ humans. The celestial compass of insects employs three or more analyzer channels. However, that compass is multimodal, i.e. e-vector information merges with directional information from other celestial cues, such as the solar azimuth and the spectral gradient in the sky, masking e-vector information. It seems that invertebrate organisms take no interest in the polarization details of visual stimuli, but polarization vision grants more practical benefits, such as improved object detection and visual communication for cephalopods and crustaceans, compass readings to traveling insects, or the alert ‘water below!’ to water-seeking bugs.

## Abstract (precis)

**Summary:** Although invertebrates are unable to see the e-vector orientation of polarized light, they can gain valuable visual information from it.

## Introduction

**Fig. 1.**
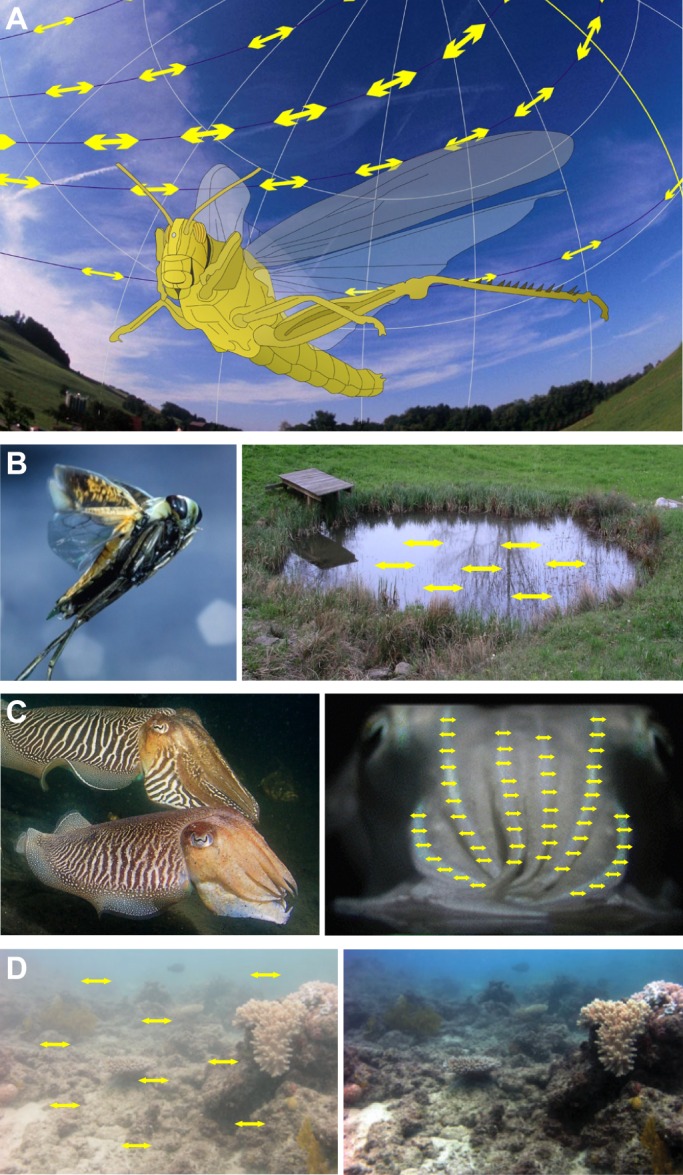
**Examples of the main functions of polarization vision in invertebrates.** (A) Polarization compass: a locust navigating by the polarization pattern of the sky. Image courtesy of Stanley Heinze. (B) Detection of water bodies: a flying backswimmer detects a pond by the horizontal polarization of light reflected from the water surface. Left panel, photograph by Kim Taylor; right panel, photograph by Thomas Labhart. (C,D) Object-based, imaging polarization vision. (C) Cuttlefish recognize the polarized body pattern of conspecifics. Left panel, photograph by Tino Brandt; right panel, photograph modified from Mäthger et al. (2009). (D) Contrast enhancement by reducing the horizontally polarized haze in the water column. The underwater scene (left) was processed using an algorithm that exploits the polarization sensitivity of photoreceptors (Schechner and Karpel, 2004) to produce an enhanced image (right). Note that this is a computer simulation demonstrating the potential gain of visibility afforded by a polarization-sensitive retina; modified from Cronin and Marshall (2011). Yellow double-headed arrows in A–D indicate the dominant e-vector orientation of partial linear polarization. All images used with permission.

The visual world provides an abundance of linearly polarized (plane-polarized; see Glossary) light stimuli hidden from the human eye, but many invertebrate organisms exploit polarized light as a source of useful visual information. Polarization vision (see Glossary) is a multi-purpose visual ability (Horvath, 2014; Marshall et al., 2011; Wehner, 2001; Wehner and Labhart, 2006), with the following proposed or actually demonstrated functions (Fig. 1). (1) Polarized skylight provides insects with a useful reference for a visual compass, which can be employed for navigation (Fig. 1A). (2) The detection of water bodies by the horizontal polarization of light reflected from their surfaces is common to many water-dependent flying insects (Fig. 1B). In the underwater world, (3) polarization patterns on the bodies of some marine animals may allow visual communication using signaling mechanisms akin to color communication (Fig. 1C), (4) polarization sensitivity can increase object contrast, thus improving general visibility (Fig. 1D), and (5) it can break luminance-based camouflage of prey or predators (not shown). All of these functions exploit the polarization of light. But that does not necessarily imply that the electric (e)-vector orientation of polarized light (see Glossary) is experienced as a separate modality of light, or that specific e-vector orientations can be perceived analogously to humans perceiving spectral stimuli as specific hues. The notion that organisms equipped with polarization-sensitive photoreceptors (see Glossary) can automatically analyze e-vector orientation is appealing, and is often assumed. For this Review, I surveyed the literature for evidence suggesting that polarization-sensitive invertebrates, or at least some species, do indeed perceive specific e-vector orientations. In some ways, this Review is also a reappraisal of the basic mechanisms of polarization vision discussed four decades ago by Bernard and Wehner (1977).

I begin this Review by defining light polarization and the basic properties of polarization vision systems. Next, I detail the rationale behind the paper, explain the technical approach and present evidence from a comprehensive literature survey. Finally, I summarize the specific properties of presently known invertebrate polarization vision systems and draw my conclusions on how polarized light is exploited by invertebrate organisms.

## Polarized light and polarization vision systems

The light emanating from the sun is unpolarized, i.e. the e-vectors of the electromagnetic waves are oriented at random. Reflection of sunlight by shiny surfaces and scattering in air and water produce partially plane-polarized light. Any plane-polarized light stimulus is defined by its e-vector orientation (φ), degree of polarization (*d*) and luminance (*l*). This definition is analogous to wavelength, spectral purity and luminance of a stimulus in the spectral domain (Bernard and Wehner, 1977; How and Marshall, 2014). Both totally polarized light (*d*=1.0) and monochromatic spectral light have maximal purity, and they consist of just one e-vector orientation or one wavelength, respectively. In partially polarized light (0<*d*<1.0) the range of e-vectors (bandwidth) is increased, and in unpolarized light (*d*=0) all e-vectors are represented equally. Again, this is analogous to broadband and white light (all wavelengths contribute equally), respectively, in the spectral domain.

**Fig. 2.**
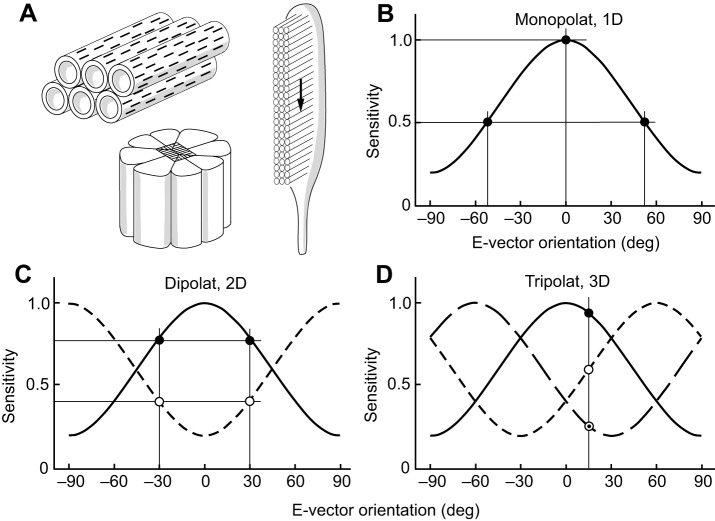
**Structural and physiological properties of polarization-sensitive invertebrate photoreceptors.** (A) Structural basis of polarization sensitivity in invertebrate photoreceptors. Right, microvilli form the rhabdomere of the photoreceptor. Light travels orthogonal to the microvilli (see arrow). Upper left, the visual pigment molecules with their elongated chromophores (black bars) reside in the microvillar membrane. Alignment of both chromophores and microvilli produces strong polarization sensitivity in the photoreceptor. Lower left, arthropod photoreceptors are grouped to form retinulae. (B–D) E-vector sensitivity functions. (B) A one-dimensional system (monopolat). (C) A two-dimensional system (dipolat) with 90 deg phase-shifted sensitivity functions. (D) A three-dimensional system (tripolat) with 60 deg phase-shifted sensitivity functions. In B–D, polarization sensitivity (PS)=5.0, degree of linear polarization (*d*)=1.0. A value of 0 deg indicates horizontal e-vector orientation and 90 deg indicates vertical e-vector orientation. Thin straight lines indicate values of selected data points (circular symbols) on the sensitivity and e-vector axes.

A plane-polarized light stimulus can be analyzed by polarization-sensitive sensors or photoreceptors, which are tuned to specific e-vector orientations. These receptors are sensitive to both e-vector orientation and degree of polarization (abbreviated to ‘degree’ hereafter). A system consisting of a single polarization-sensitive receptor [a one-dimensional or 1D (monopolatic) system; see Glossary; Fig. 2B] is polarization-blind (in analogy with color-blindness in monochromats), because, by adjusting light intensity or degree, different e-vectors can elicit identical responses in the photoreceptor (owing to the principle of univariance). Thus, in the monopolat represented in Fig. 2B, a 0 deg stimulus of a given light intensity will elicit the same response as a −52 or +52 deg stimulus of doubled intensity.

List of abbreviations

1D, 2D or 3D system: one-, two- or three-dimensional polarization vision system
deg: angular degree
degree: degree of polarization
DRA: dorsal rim area of insect compound eye
e-vector: e-vector orientation
PS: polarization sensitivity

**Fig. 3.**
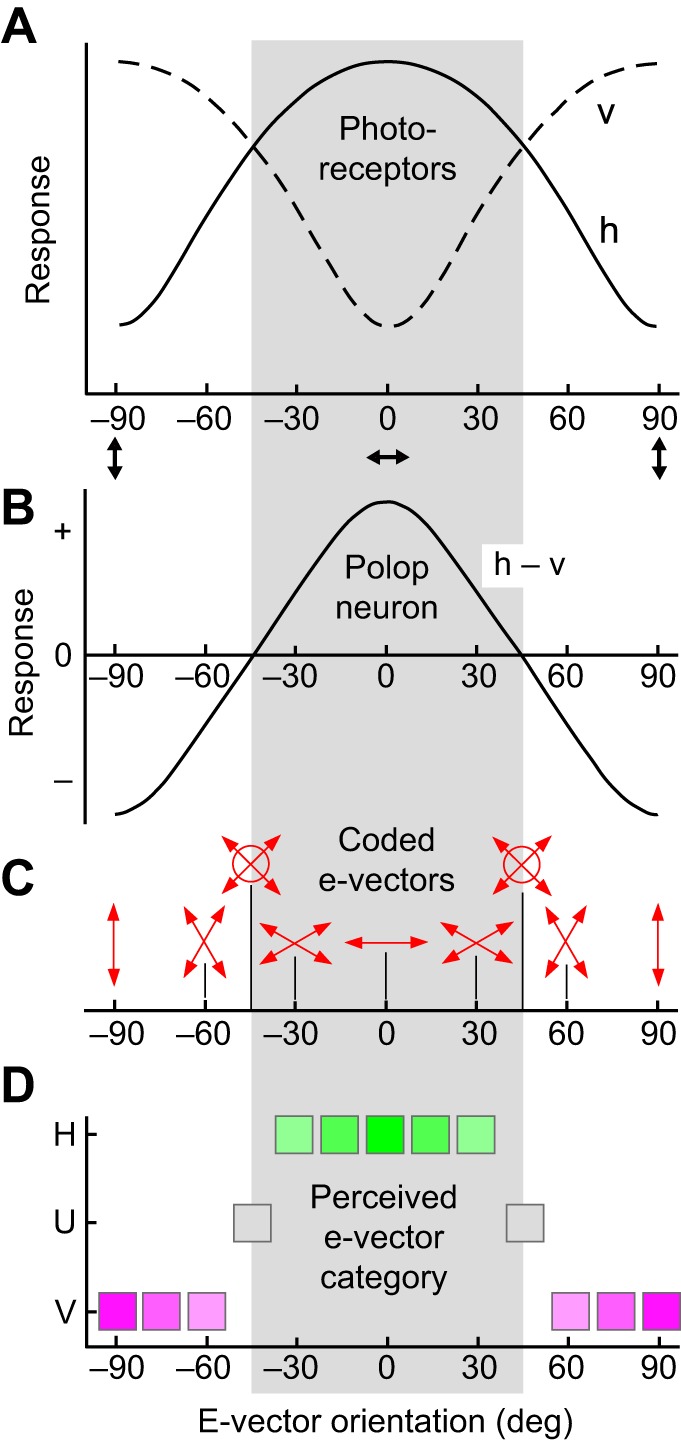
**E-vector coding properties of an orthogonal dipolatic (2D) system.** (A) E-vector response functions of photoreceptors with horizontal (h) and vertical (v) e-vector tuning axes. (B) E-vector response function of a polarization-opponent (polop) neuron (h−v). (C) E-vector coding by a polop neuron. For a defined degree of polarization (e.g. *d*=1.0), coding is ambiguous for all e-vectors (double-headed arrows) except for 0 deg and ±90 deg. E-vectors of ±45 deg are also confused with unpolarized light (indicated by circles). For undefined degrees of polarization, not just two but an infinite number of e-vectors can produce the same response in the polop neuron, all appearing identical to a dipolat. (D) The two perceptual categories ‘horizontal’ (H; green symbols) and ‘vertical’ (V; purple symbols) of e-vector orientation perceived by the dipolat. Excitation or inhibition of the polop neuron elicits perception of either H or of V, respectively. Intensity of color indicates perceived saturation. Stimuli of ±45 deg are perceived as unpolarized light (U; gray symbols).

**Fig. 4.**
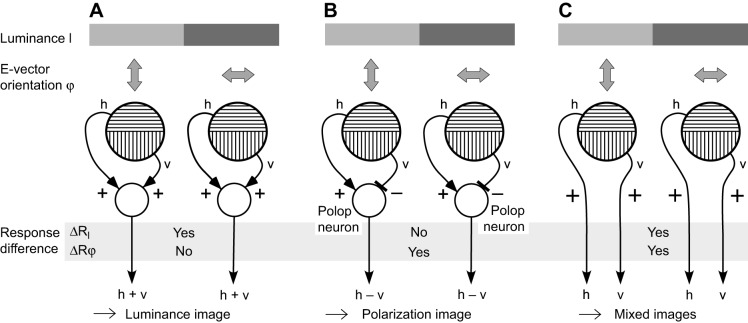
**Effects of interactions between the signals of polarization-sensitive photoreceptors in an orthogonal dipolatic system.** (A–C) Two ommatidia view a visual contrast of both luminance (gray bars) and e-vector orientation (double-headed arrows). h and v are the responses of photoreceptors with horizontal and vertical e-vector tuning axes, respectively. Plus and minus signs mark activating and inhibiting connections, respectively. ΔR_I_ and ΔR_φ_ show the difference between the responses of the two ommatidia for luminance contrast and e-vector contrast, respectively. (A) Pooling h and v provides a largely polarization-insensitive luminance image. For receptors with high PS, the pooled signal retains some e-vector dependence because of the logarithmic relation between photon absorption and photoreceptor response (apparent in the log-cos^2^-shaped e-vector response functions of the photoreceptors of Fig. 3A). (B) Polarization-opponent interaction between h and v by polop neurons produces a luminance-independent polarization image. (C) Keeping h and v separate provides images that are sensitive to both polarization and luminance.

Obviously, a single receptor does not suffice to analyze e-vector orientation; instead, the signals of different receptor types (analyzer channels), tuned to different e-vectors, must be compared. In a two-dimensional system [2D (dipolatic) system; Fig. 2C], the signals of two analyzer channels are compared by a polarization-opponent comparator neuron (a polop neuron, see Glossary; Figs 3B, 4B) (Bernard and Wehner, 1977; How and Marshall, 2014). However, a 2D system is unable to determine e-vector orientations unequivocally. This is obvious for a totally polarized stimulus that is analyzed by an orthogonal 2D system (Fig. 2C); any pairs of e-vectors equidistant from 0 deg (e.g. ±30 deg) elicit identical responses in the two photoreceptors; consequently, the two e-vectors are undistinguishable. Polarization-sensitive receptors also respond to degree, i.e. the smaller the degree, the weaker the modulation of the e-vector response function. Therefore, for a polarized stimulus of unknown degree (as under natural conditions), an infinite number of e-vectors produce identical receptor and polop responses, thus appearing identical to a dipolat. In other words, 2D polarization vision has so-called confusion states (Bernard and Wehner, 1977), formally defined by a ‘polarization distance’ of 0 (How and Marshall, 2014). However, dipolats can safely discriminate two ranges of e-vector orientation, e.g. horizontal (H; 0 deg±<45 deg), indicated by a depolarization, and vertical (V; 90 deg±<45 deg) signaled by a hyperpolarization of the polop neuron (Fig. 3). The polop neuron is unresponsive to both ±45 deg stimuli and to unpolarized light and, therefore, these three stimuli are confused. For all these reasons, 2D polarization vision is unsuitable for subtle e-vector analysis. For a more formal discussion of 2D polarization vision systems, see How and Marshall (2014).

Note that dipolatic polarization vision is analogous to dichromatic color vision, which recognizes just two categories of hues (‘short wave’ versus ‘long wave’; Neitz et al., 2001; Vienot et al., 1995), and which confuses a specific monochromatic light (e.g. 480 nm in dogs; Neitz et al., 1989) with white light. For a discussion of further analogies between polarization and color vision, see Bernard and Wehner (1977).

Theory shows that in order to analyze e-vector orientation unequivocally, the signals of at least three analyzer channels with different e-vector tuning axes (see Glossary) must be compared [i.e. a three-dimensional or 3D (tripolatic) system is required; Bernard and Wehner, 1977; Kirschfeld, 1972]. Thus, each e-vector orientation is unequivocally coded by a signal triplet provided by the three analyzer channels (exemplified in Fig. 2D for a 15 deg e-vector). Both the e-vector orientation of totally plane-polarized light (*d*=1.0) and the dominant e-vector orientation of partially plane-polarized light can be extracted from the signal triplet mathematically (Bernard and Wehner, 1977). This notion was successfully tested with mobile robots navigating by polarized skylight, evaluating the signals of three artificial polarization sensors by means of mathematical algorithms (Lambrinos et al., 1997; Lambrinos et al., 2000). Using computer modeling, the signal triplet can also be analyzed by an artificial neural network (Sakura et al., 2008). In addition, wiring diagrams for evaluating the signals of the three analyzer channels have been proposed (Bernard and Wehner, 1977; How and Marshall, 2014).

Glossary **E-vector orientation**

The orientation of the electric field of electromagnetic waves, which oscillates orthogonally to the direction of wave propagation.

**E-vector perception**

Perception of specific e-vector orientations, which includes the perception of plane-polarized light as a separate modality of light.

**E-vector tuning axis**

The e-vector of plane-polarized light eliciting maximal depolarization or spike frequency in a polarization-sensitive photoreceptor or neuron.

**Monopolat, dipolat, tripolat**

Organism equipped with a one-, two- or three-dimensional polarization vision system.

**One-, two- or three-dimensional polarization vision system**

Polarization vision system receiving input from one, two or three polarization-sensitive analyzer channels with different e-vector tuning axes. I also use the expressions monopolatic, dipolatic and tripolatic for these systems.

**Plane-polarized light**

Also called linearly polarized light. Light in which the distribution of e-vector orientation is not random but exhibits a dominant orientation (partially plane-polarized), or in which all e-vectors have the same orientation (totally polarized light).

**Polarization-sensitive photoreceptors or neurons**

Photoreceptors or neurons in which electrical activity is a function of the e-vector orientation of plane-polarized light.

**Polarization vision**

In this Review, I use a wide definition of the term. It means any visual perception that is based on polarization-sensitive photoreceptors. Accordingly, even monopolats have some sort of polarization vision, i.e. about the same as a human observer looking through a polarizing filter. The specific quality of the term ‘polarization vision’ is defined by the context in the paper.

**Polop neurons**

Neurons receiving opponent input from two polarization-sensitive analyzer channels with different e-vector tuning axes; specifically, input from two photoreceptor populations with orthogonal microvilli orientations in individual ommatidia.

**Rhabdomere twisting**

Instead of remaining constant (as in Fig. 2A), the orientation of the microvilli changes continuously along the rhabdomere. Twisting can involve the whole retinula, including the cell bodies of the photoreceptors.

**True polarization vision**

Perception of polarization (e-vector, e-vector category) as a separate modality of light, i.e. not influenced by the luminance or spectral composition of a plane-polarized light stimulus.

## Requirements for unambiguous and unbiased e-vector detection

I reason that unambiguous analysis of e-vector orientations as provided by three- or higher-dimensional (multidimensional) polarization vision systems is a prerequisite for perceiving specific e-vector orientations (hereafter referred to as ‘e-vector perception’; see Glossary), analogous to human perception of spectral stimuli as specific colors. In other words, if the result of an e-vector analysis is ambiguous, e-vector orientation cannot be perceived as a unique perceptual attribute of a physical (dominant) e-vector orientation. Because of their ambiguities, 2D and 1D systems are insufficient for e-vector perception. Please note that in the present context, the term ‘perception’ does not necessarily imply a conscious act. Rather, it means the ability to extract unambiguous e-vector orientation as an attribute of a visual stimulus.

Apart from three-dimensionality, four more criteria must be fulfilled for unbiased e-vector perception. (1) All three analyzer channels must share a common visual field, i.e. they must view one and the same stimulus. (2) The system must be monochromatic, i.e. all analyzer channels should have the same spectral sensitivity. This makes it insensitive to the spectral composition of a stimulus and avoids interference with and confusion between spectral and polarizational components. (3) The system must be insensitive to the luminance of the stimulus. In imaging, object-based polarization vision systems, the polarization image must be independent of the luminance-based image. If the polarization sensitivity (PS) of the photoreceptors merely serves to enhance luminance contrast (image enhancement; Fig. 1D) (as discussed by How et al., 2015; Johnsen et al., 2011), polarization information is lost and PS merely has a helper function. In this case, the responses of the polarization-sensitive photoreceptors provide mixed luminance/polarization images (Fig. 4C). (4) Processing of the analyzer signals must be of the ‘simultaneous’ type, i.e. all three analyzers have to cooperate simultaneously by comparing their outputs (Kirschfeld, 1972). In the ‘successive’ mode of e-vector analysis (Kirschfeld, 1972), in which one analyzer rotates about its optical axis, successive readings of the analyzer response are compared. Maximal response indicates that the receptor's e-vector tuning axis is aligned with the e-vector of the stimulus. The successive mechanism of e-vector analysis is a multi-step process, which involves additional proprioceptive and/or visual flow information (Wehner and Labhart, 2006); therefore, I consider it an unlikely option for providing e-vector perception.

The term ‘true polarization vision’ (see Glossary) is sometimes used to express the ability of an organism to perceive polarization as a separate modality of light, i.e. independent of stimulus luminance and spectral composition (Nilsson and Warrant, 1999; Schwind, 1984). To achieve this, those photoreceptor response components that are due to luminance and spectral composition of a stimulus have to be separated from the component produced by light polarization. This is readily obtained by opponent interaction between the signals of two homochromatic photoreceptors with orthogonal e-vector tuning axes, i.e. by homochromatic polop neurons. Thus, a 2D system already suffices for true polarization vision, in that different e-vector ranges or categories may be perceived as a separate modality of light. However, to identify specific e-vector orientations (e-vector perception), an additional analyzer channel (≥3D system) is required. The term ‘e-vector perception’ automatically implies true polarization vision.

## How can the presence of e-vector perception be assessed?

The presence of e-vector perception in an organism could, in principle, be demonstrated by behavioral experiments. However, this is extremely difficult to accomplish. First, one has to prove that the organism is able to identify specific (dominant) e-vector orientations independent of luminance and degree, similar to a trichromat identifying specific hues independent of luminance and spectral purity. Second, one has to make sure that a dedicated polarization vision path exists that operates independently of luminance and color perception. Thus, the behavioral demonstration of e-vector perception is a formidable task, which so far no one has attempted.

Instead of trying to prove e-vector perception behaviorally, a more practicable approach is to study the dimensionality of polarization vision systems. As discussed above, the number of polarization-sensitive channels determines whether e-vector perception is possible, in principle, or must be excluded. How can the dimensionality of polarization vision systems be assessed? PS in invertebrate photoreceptors is based on the absorption properties for polarized light of the microvilli that form the rhabdomeres, the light-sensitive structure of invertebrate photoreceptors (Fig. 2A). By still little-known mechanisms (Roberts et al., 2011), the chromophores of the visual pigment molecules are aligned within the microvillar membrane (Fig. 2A) in such a way that plane-polarized light is maximally absorbed when the e-vector orientation of a stimulus is parallel to the long axis of the microvilli (Goldsmith and Wehner, 1977; Hardie, 1984, 1985; Israelachvili and Wilson, 1976; Kirschfeld, 1969). Therefore, microvilli orientation is a convenient indicator of the e-vector to which a photoreceptor is tuned.

To assess the dimensionality of a polarization vision system, the microvilli orientation of the relevant photoreceptors can be measured histologically, usually by electron microscopy (e.g. see Wernet et al., 2012). When doing this, one has to make sure that the microvilli are reasonably aligned along the length of the rhabdomere (Fig. 2A), because misalignment caused by rhabdomere twisting (see Glossary) or random misalignment would reduce or even abolish PS, and would strongly affect the e-vector tuning axis (Nilsson et al., 1987; Wehner et al., 1975; Wernet et al., 2012). If the identity of the involved photoreceptors is unknown, the number of microvillar types within an ommatidium or within a retina can still indicate the highest possible dimensionality. Electrophysiology may also provide dimensionality information, namely when discrete groups of photoreceptors or neurons with different e-vector tuning axes are found.

Once the presence of a multidimensional system is established, it must be scrutinized for the secondary criteria (points 1 to 4 in the previous section), based on additional behavioral or electrophysiological data. Fulfillment of all necessary requirements indicates a potential for e-vector perception; however, it does not prove its implementation. In this Review, I will therefore not attempt to prove e-vector perception, but using a *reductio ad absurdum* approach I will test whether e-vector perception is possible, in principle, in any of the presently known invertebrate polarization vision systems.

Although there is a host of studies providing data on microvilli orientation in invertebrate retinae, I will mainly focus on those species both for which relevant data on retinal anatomy are available, and in which behavioral responses to polarized light have been studied. This restriction is necessary because anatomical or electrophysiological evidence of photoreceptor PS alone is insufficient to prove that polarized light information is actually exploited by an organism. Below, I review the literature in search of evidence suggesting the existence of e-vector perception in invertebrates.

## Searching for evidence of e-vector perception

### Two-dimensional systems

The input stages of all assumed and actually proven invertebrate polarization vision systems studied were found to be dipolatic, receiving input from photoreceptors with mutually orthogonal microvilli orientations (Fig. 2C, 5A,B top rows) (Bernard and Wehner, 1977; Dacke, 2014; Horvath and Varju, 2004; Labhart and Meyer, 1999; How and Marshall, 2014; Marshall and Cronin, 2014; Wehner and Labhart, 2006; Zeil et al., 2014). As discussed above, 2D systems can identify two broad e-vector ranges, but because of ambiguities and the confusion of e-vector and degree, the detection of specific e-vector orientations is impossible. Considering their limited e-vector analyzing properties, one might ask how dipolatic systems may be useful to an organism, if at all. Below, I show that 2D systems can execute a variety of important functions in spite of their restrictions.

**Fig. 5.**
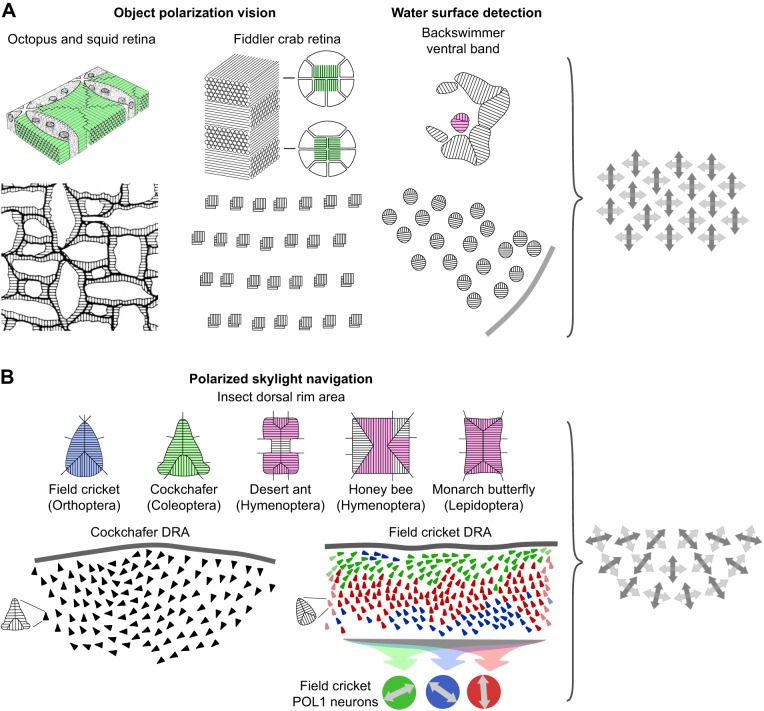
**Overview of the basic sensory principles in invertebrate polarization vision.** (A) Two-dimensional (dipolatic) polarization vision. (B) Multidimensional (polypolatic) polarization vision with dipolatic input stages. Upper rows in A and B: rhabdoms of photoreceptors providing polarization vision and their microvilli orientations. Colors indicate the spectral receptor types of the involved photoreceptors; purple indicates UV sensitivity. Note that all input stages are dipolatic. Lower rows in A and B: retinal arrays of ommatidia and photoreceptors. Up is dorsal. Note the characteristic differences between the arrays in A (aligned) and those in B (fan-like). The cricket DRA in B (lower row) is represented in part only. The three cricket POL1 neurons (colored circles in B, lower row) receive input from DRA ommatidia as indicated by the colors; double-headed arrows denote their e-vector tuning axes. Right in A and B: schematic representations of the two types of retinal array, i.e. aligned versus fan-like arrangement of input stages. Crossed double-headed arrows indicate the e-vector tuning axes of dipolatic input stages. Diagrams are not to scale. Cephalopod schematics are modified from Moody and Parriss (1961) and Saidel et al. (1983), with permission. DRA ommatidia are reproduced from Wehner and Labhart (2006), with permission. Other images are after Alkaladi et al. (2013), Labhart et al. (1992, 2001), Schwind (1983) and Waterman and Horch (1966).

#### Detection of water bodies by flying, water-dependent insects

A number of flying, water-dependent insects, including dragonflies and the backswimmer *Notonecta*, detect water bodies by the horizontal e-vector of light reflected from their surfaces (Horvath and Csabai, 2014; Lerner, 2014; Schwind, 1984, 1991; Wildermuth, 1998) (Fig. 1B). These insects only have to detect strong horizontal polarization in the ventral field of view, and exact e-vector analysis is not required. In this case, a simple detector system with opponent input from just two horizontally and vertically tuned analyzer channels suffices. In the retina of the backswimmer, the photoreceptors representing the two channels can actually be observed (Fig. 5A, right) (Schwind, 1983, 1984).

#### Object-based, imaging polarization vision in cephalopods and crustaceans

Coleoid cephalopods (octopods, squid and cuttlefish) keep their eyes or heads at a constant orientation (Talbot and Marshall, 2011). Their retina contains two types of blue/green-sensitive photoreceptors with either horizontally or vertically oriented microvilli (Mäthger et al., 2009; Saidel et al., 1983; Talbot and Marshall, 2011) (Fig. 5A, left). Squid and cuttlefish show improved prey catching when a polarization contrast between background and prey is provided (Cartron et al., 2013; Shashar et al., 1998). In addition, some cephalopods are able to produce distinct polarization patterns on their bodies, which may facilitate visual communication (Fig. 1C) (Cronin et al., 2003; Shashar et al., 1996). Cephalopods can detect patterns consisting of polarization contrast alone, i.e. without any luminance contrast (Moody and Parriss, 1961; Pignatelli et al., 2011; Shashar and Cronin, 1996; Shashar et al., 1996; Temple et al., 2012). Experiments with cuttlefish suggest that there are separate pathways for analyzing luminance-contrast and polarization-contrast patterns (Cartron et al., 2013). Interestingly, cuttlefish detect looming polarized stimuli with e-vector contrasts of just a few deg to the background, in spite of their 2D system (Temple et al., 2012).

In crustaceans, a typical ommatidium contains two sets of blue/green receptors (R1–7) with untwisted rhabdoms and mutually orthogonal, horizontally and vertically oriented microvilli (Fig. 5A, middle) (Marshall and Cronin, 2014). This is the same arrangement as in the cephalopod retina. However, the situation in several crustaceans, and especially in stomatopods, is more complex (see Box 1). But so far, none of the crustacean retinae studied provides a robust basis for multidimensional (>2D) polarization vision.

Box 1. Evaluation of potential multidimensional polarization vision systems in crustaceans

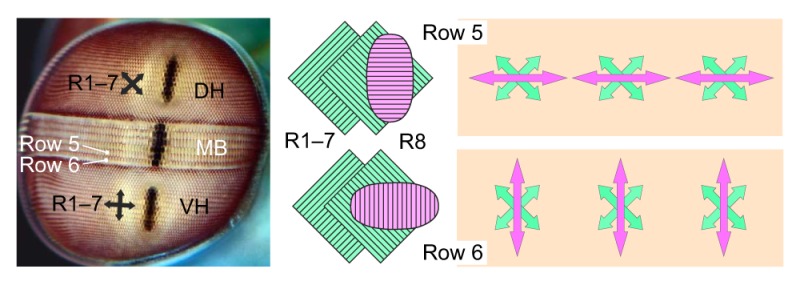

In addition to the full-sized R1–7 receptors, a short distally positioned UV/violet receptor (R8) may be present, containing misaligned or bi-directional microvilli throughout much of the retina (Marshall et al., 1991; Marshall and Cronin, 2014). But polarization-sensitive R8 cells with parallel microvilli at 45 deg to the orthogonal e-vector tuning axes of R1–7 were found in the ommatidia of rows 5 and 6 of the mid-band (MB) of the mantis shrimp eye, and these might potentially form a third analyzer channel (see photograph and schematic rhabdoms; photograph by Roy Caldwell; diagrams based on Marshall, 1988). However, a tripolatic retinal system consisting of R1,4,5 versus R2,3,6,7 versus R8 is unsuitable for unbiased e-vector analysis, because the R1–7 cells are blue/green-sensitive while R8 cells, including those of rows 5 and 6, are UV-sensitive, making the system heterochromatic (green versus purple rhabdomeres). But because R8 microvilli are orthogonal between row 5 and row 6, a separate, dipolatic UV polarization vision system across the two rows is possible (purple arrows; Kleinlogel and Marshall, 2005, 2006). Behavioral evidence of UV polarization sensitivity is required to confirm this hypothesis. In some species, the rhabdom of R8 in MB rows 5 and 6 acts as a quarter-wave retarder, converting the R1–7 cells of these rows to circular polarization analyzers (Chiou et al., 2008).

The dipolatic R1–7 systems in the dorsal (DH) and ventral hemispheres (VH) of stomatopod eyes are rotated 45 deg to each other (see crossed double-headed arrows in photograph; Marshall and Cronin, 2014; Marshall et al., 1991). With their strongly overlapping visual fields (note pseudopupils) they could cooperate at higher levels, forming a 4D system, but this has not yet been tested by behavioral experiments.

In crayfish, the e-vector tuning axes of photoreceptors near the dorsal eye rim were reported to cluster around three orientations (Glantz, 2007), potentially forming a 3D system. However, in crayfish, PS seems to support optokinetic and defense reflexes (Glantz, 2008) by enhancing image contrast, and evidence for e-vector guided navigation is missing so far.

Stomatopods are the only crustaceans so far shown to be able to learn e-vector orientations independent of luminance (Marshall et al., 1999). Like cuttlefish, fiddler crabs respond to looming polarized stimuli with e-vector contrasts of just 3.2 deg to the background, in the absence of luminance contrast (How et al., 2012). Looming polarized stimuli were also used to test whether the responses of fiddler crabs were compatible with a 2D system of polarization vision (How et al., 2014). In that study, stimulus and background had the same e-vector but differed in degree. The data confirm the expectation that the crabs can discriminate between different degrees of polarization. Stimulus detection was strongly impaired when the e-vectors of stimulus and background were at +45 deg and −45 deg to the horizon, respectively, which agrees with an expected null point of discrimination of a horizontal/vertical 2D system (Bernard and Wehner, 1977; How et al., 2014). The stomatopods also tested in that study for comparison (How et al., 2014) did not exhibit a null point, which is not unexpected considering their various separate and differently oriented 2D systems (Box 1). Alternatively, they could have used a successive approach based on eye stalk rotation (Daly et al., 2016; Land et al., 1990).

At first glance, two of the behavioral studies presented above seem to contradict the concept of 2D polarization vision, which excludes e-vector perception: both cuttlefish and fiddler crabs detect looming stimuli with e-vectors differing by just a few deg from the background e-vector (How et al., 2012; Temple et al., 2012). How can this performance be explained? Although 2D systems lack the ability of unambiguous e-vector analysis, they still allow the discrimination of stimuli with different states of polarization, involving e-vector and/or degree. To illustrate this, consider a polarized stimulus of one e-vector on a background of another e-vector but with the same degree and luminance (e-vector contrast). Whenever the photoreceptor responses to stimulus and background differ by at least a threshold amount because of the e-vector difference, the stimulus will be detected, although the two e-vectors cannot be perceived.

Alternatively, consider a polarized stimulus of a certain degree on a background of another degree but with the same e-vector and luminosity (degree contrast). Because the photoreceptors are sensitive to both e-vector and degree, the stimulus will again be detected whenever the difference between the photoreceptor responses reaches a threshold. Of course, the same applies when there is contrast in both e-vector and degree. The stimulus becomes undetectable only if the polarization distance between stimulus and background dips below threshold, i.e. if stimulus and background produce (almost) the same photoreceptor responses (How and Marshall, 2014). Thus, although cuttlefish and fiddler crabs are poor at absolute e-vector analysis, paradoxically they can detect even minute e-vector differences. Generally speaking, dipolats are unable to see e-vector orientations, but they can perfectly well detect e-vector contrasts.

Although cephalopods and crabs cannot perceive specific e-vector orientations, they do have polarization vision, in the sense that they can discriminate between different plane-polarized stimuli of the same luminance. However, their dipolatic polarization sense perceives just two categories of e-vectors (Fig. 3D), analogous to dichromats perceiving two categories of hues. Both dipolats and dichromats can assign any stimulus to one of the two categories, and they can discriminate different stimuli within each category. However, they are unable to disentangle stimulus quality (e-vector, hue) and stimulus purity (degree of polarization, spectral purity) within a given category. This coarse, categoric e-vector analysis is probably just one function of the orthogonal 2D systems of cephalopods and crustaceans. A more important function may be signal conditioning by the polop neurons, providing enhanced polarization contrast and luminance independence, in a similar way as in the insect polarized skylight compass (discussed in detail below).

#### Detectors for polarized skylight in insects and spiders

A system exploiting skylight polarization for navigation must necessarily be able to evaluate the directional component of polarized light, i.e. e-vector orientation. In insects, polarized skylight navigation is mediated by the specialized dorsal rim area (DRA) of the compound eyes. The individual ommatidia of all DRAs studied so far are orthogonally dipolatic (Fig. 5B, top row) (reviewed in Dacke, 2014; Heinze, 2014a; Labhart and Meyer, 1999; Wehner, 2014; Wehner and Labhart, 2006; Zeil et al., 2014; and recently reported in Fischer et al., 2014; Wernet et al., 2012; Yamahama et al., 2014). Because the dipolatic ommatidia of the DRA are unfit for exact e-vector analysis, they are believed to function in signal conditioning: owing to the antagonistic processing of the photoreceptor signals, the polop neurons act as differential polarization sensors (Labhart and Meyer, 2002) (Figs 3B, 4B). These effectively enhance polarization contrast and simultaneously make the system insensitive to the variations in absolute light level (Labhart and Meyer, 2002; Labhart et al., 2001). As discussed below, the polop neurons of the DRA, in turn, provide the input to the actual e-vector analyzing system.

Conditions that are functionally analogous to the insect DRA were found in the camera-type eyes of some spiders. Here, the upwards-directed ventral retina of certain eye types also contains two photoreceptor populations with mutually orthogonal microvilli orientations (Dacke et al., 2001; Mueller and Labhart, 2010). The spider *Drassodes* dedicates a complete eye pair to polarized skylight detection, again forming an orthogonal 2D system (Dacke et al., 1999).

### Three- and higher-dimensional systems

#### The ommatidial array of insect dorsal rim areas

As demonstrated for several insect orders, the dipolatic ommatidia forming the insect DRA are arranged in a characteristic fan-like fashion (Fig. 5B) (reviewed by Heinze, 2014a; Wehner and Labhart, 2006; Zeil et al., 2014). Correspondingly, the e-vector tuning axes of the polop neurons change gradually across the DRA, forming a potential multidimensional system. For proper e-vector analysis, the visual fields of the DRA ommatidia must coincide or at least overlap to a large extent, i.e. the ommatidia must receive light from the same part of the sky. Because of both a restriction of the DRA to the dorsal-most eye part and strongly enlarged visual fields of the ommatidia, this condition is indeed met in some insects, such as crickets, locusts and cockchafers (Fig. 5B) (Blum and Labhart, 2000; Labhart et al., 1992; Schmeling et al., 2015). By comparing the output signals of the differently tuned polop neurons, such DRAs could, in principle, determine e-vector orientation within their common visual field. However, the actual neural algorithms used to extract e-vector orientation from the multiple polop signals require further investigation. As demonstrated by behavioral laboratory experiments, crickets and locusts can indeed analyze e-vector orientation with their DRAs (Brunner and Labhart, 1987; Mappes and Homberg, 2004). But, as will be elucidated in detail in the context of the cricket POL1 neuron system, celestial compasses are visuo-multimodal systems and are not driven by polarized skylight alone, but they receive input from multiple celestial cues, a property that is incompatible with e-vector perception.

In some insects, such as *Drosophila* (Weir et al., 2016; Wernet et al., 2003), monarch butterflies (Labhart et al., 2009; Stalleicken et al., 2006) and desert ants (Labhart, 1986; Wehner, 1982), the DRA forms a narrow band along the dorsal eye margin. The ommatidia have small acceptance angles and diverging optical axes, keeping their visual fields separated. Apparently, such DRAs are not designed to evaluate local e-vector orientations, but they must somehow exploit the combined polarization signals from different parts of the celestial polarization pattern.

#### The three-dimensional POL1 neuron system of crickets

Although the cricket DRA is also multidimensional, a 3D system exists at the level of the optic lobe. It consists of three e-vector types of polarization-opponent neurons (POL1 neurons; tuned to ∼10, 60 and 120 deg versus the head length axis) receiving opponent input from the orthogonally dipolatic ommatidia (Labhart et al., 2001). Apparently, at the optic lobe level, the retinal multidimensionality is reduced to three analyzer channels by neural integration (Labhart et al., 2001). Conforming to all four of the secondary criteria outlined above (see Requirements for unambiguous and unbiased e-vector detection), (1) the visual fields of the three e-vector types of POL1 neurons are practically identical (Labhart et al., 2001). (2) The bulk of the rhabdom formed by the principal receptors contains a blue-absorbing visual pigment; just 1% of the visual pigment, contained in the small proximal R8 cell, is a UV pigment (Blum and Labhart, 2000; Henze et al., 2012). Thus, the DRA is practically monochromatic, a view that is supported by the spectral sensitivity of POL1 neurons (Labhart and Petzold, 1993). (3) The polarization opponency of POL1 neurons makes the system insensitive to the variations of absolute light level (Labhart, 1988). And (4), cricket polarization vision operates in the simultaneous mode, as demonstrated by behavioral tests (T.L., unpublished observations). Thus, the POL1 system seems to fulfill all necessary conditions for e-vector perception.

However, the following two caveats must be considered. First, the cricket polarization compass is a non-imaging, wide-field visual sub-system that integrates over a wide area of sky (Labhart et al., 2001; Wehner and Labhart, 2006). This excludes the analysis of the individual e-vectors composing the celestial polarization pattern, but it would allow perception of average e-vector orientation within the field of view. Second, and more importantly, polarization-sensitive neurons in the brains of locusts, monarch butterflies and dung beetles also respond to unpolarized stimuli in a wavelength- and/or position-dependent way. This suggests that directional information from the chromatic and intensity gradients of the sky and from the sun also contribute to the celestial compass (el Jundi et al., 2015; reviewed by el Jundi et al., 2014a; Heinze, 2014a,b; Pfeiffer and Homberg, 2007), possible influences that have not been studied in the cricket. The visuo-multimodality of celestial orientation was also demonstrated by behavioral experiments in bees (Rossel and Wehner, 1984; Dyer and Gould, 1983), desert ants (Wehner, 1997; Wehner and Müller, 2006), monarch butterflies (Reppert et al., 2004) and dung beetles (Dacke et al., 2014; el Jundi et al., 2015, 2014b); celestial orientation involves the solar azimuth (bees, desert ants, monarchs, dung beetles), the spectral gradient in the sky (bees, ants, dung beetles) and the intensity gradient (dung beetles), in addition to skylight polarization. In desert ants, the polarization compass seems to work independently of the sun compass (Wehner and Müller, 2006) but, as explained above, the ant's DRA does not qualify for e-vector perception for optical reasons (Labhart, 1986; Wehner, 1982).

Thus, although the e-vector detection system of the cricket is monomodal, the celestial compass into which it feeds is probably multimodal by analogy with other insects. Therefore, e-vector perception by crickets can hardly be expected. This argument may be neglected under certain laboratory conditions where all directional information is restricted to e-vector orientation, such as for a polarized monochromatic stimulus presented in the zenith (Henze and Labhart, 2007). For a discussion of the question of what a cricket may actually see in such a situation, see below.

#### The ocellar system of the orchid bee

In addition to the compound eyes, most insects are equipped with three small single-lens eyes, called ocelli. Ocellar photoreceptors are polarization-sensitive in some species (Geiser, 1985; Geiser and Labhart, 1982; Mote and Wehner, 1980). This is due to the sheet-like shape of their rhabdomeres and the corresponding alignment of the microvilli (reviewed by Zeil et al., 2014). As in other hymenopterans, in the orchid bee, the orientation of the ocellar rhabdoms shows a monomodal distribution (Taylor et al., 2016; Zeil et al., 2014). However, unlike the ocelli of other hymenopterans, the visual fields of the three ocelli are not completely separated, but exhibit a wide dorsal overlap (Geiser, 1985; Taylor et al., 2016). In addition, because of their different orientations, the ocelli have strongly diverging e-vector tuning axes. Thus, the orchid bee's ocelli may form the input channels of a 3D polarized skylight navigation system. Note that, as an exception, the input stages of this system, the ocelli, are monopolatic instead of dipolatic. It still remains to be demonstrated by behavioral experiments that the orchid bee's ocelli serve indeed as a polarization compass, and if they complement the compound-eye-based celestial compass or operate as a separate system, for instance, at low light levels (Taylor et al., 2016).

#### The honey bee retina

In honey bees, the twisted retinulae of the regular ommatidia (ventral to the DRA) abolish PS of the long UV photoreceptors (Labhart, 1980; Wehner et al., 1975). But the short, proximal UV receptor R9 is little affected by the twist and remains polarization-sensitive. Neighboring ommatidia twist in opposite directions, producing two e-vector tuning types of R9. Together with the long, polarization-insensitive UV receptors, they could theoretically form a 3D system, in which one analyzer channel, represented by the polarization-insensitive long UV receptors, measures light intensity (for details, see Wehner et al., 1975). However, so far, behavioral experiments with bees have produced no evidence for polarization vision outside the DRA (Foster et al., 2014; Lau, 1976; Wehner and Strasser, 1985; K. Pfeiffer, University of Marburg, personal communication).

In light of the previous argument, would a third, polarization-insensitive channel solve the ambiguity problem of the previously discussed orthogonal dipolats? A separate intensity channel could be gained by pooling the responses of the two photoreceptors already present (Fig. 4A). Unfortunately, such a system works only with non-orthogonal, polarization-sensitive channels (Wehner et al., 1975), which definitely excludes e-vector perception for the orthogonally dipolatic cephalopods, crustaceans and backswimmers, and by individual ommatidia of insect DRAs.

In contrast to Wehner et al. (1975), Ribi (1980) reported three microvillar types of R9 in neighboring ommatidia of the dorsal, non-DRA part of the eye, which could potentially form a 3D system. However, Wehner and Strasser (1985) found that orientation responses were abolished after painting out the DRA, indicating that the unspecialized dorsal eye was polarization-insensitive.

#### Polarization vision in the butterfly *Papilio*

Unlike in bees, the photoreceptors of *Papilio* have retained moderate PS in the whole eye (Kelber et al., 2001). Each ommatidium contains photoreceptors with four different microvilli orientations, forming the basis for a 4D system (Kelber et al., 2001). Both feeding and ovipositing *Papilio* can be trained to different e-vectors (Kelber et al., 2001). However, the ommatidia are not just tetrapolatic but also polychromatic, making them unfit for e-vector perception (Kelber et al., 2001). Behavioral tests show that spectral and polarization components of stimuli are not processed separately (Kelber et al., 2001). This seemingly confused multi-input system may have an ecological significance, namely to enhance the attractivity of horizontally oriented green leaves during egg-laying, which offer better protection for the eggs than vertically oriented leaves (Kelber et al., 2001). Interestingly, different e-vector orientations in homochromatic stimuli are not perceived as different colors but as brightness differences, indicating that polarization information is lost at the perception level (Kinoshita et al., 2011). The *Papilio* case is a good example showing that polarization sensitivity can have just a helper function subserving other visual tasks.

#### Ventral polarization sensitivity in *Drosophila*

An alignment response to ventrally presented polarized light has been detected in *Drosophila*, demonstrating PS in the downward-looking part of the eye (Wernet et al., 2012). Unlike in the DRA, rhabdomere twisting is not absent in the ventral receptors but is generally moderate enough to allow a useful level of PS. UV-sensitive R7p cells and some of the outer, blue/green-sensitive receptors (probably R4–R6) seem to be involved; blue- and green-sensitive R8 cells (R8p and R8y, respectively) possibly also contribute to the response. Thus, although the different receptor types exhibit more than two e-vector tuning axes (T.L., unpublished observations), the lack of monochromacy prohibits e-vector perception.

## Taking stock of invertebrate polarization vision

### Object-based polarization vision

The object-based, imaging polarization vision systems of cephalopods and crustaceans are dipolatic and unfit for precise e-vector analysis. Why have mechanisms for tripolatic e-vector analysis not developed in these visual systems? As in color vision, a third polarization-sensitive channel would vastly increase the information capacity of polarization vision (Neitz et al., 2001). In insects, object-based polarization vision has only been demonstrated in *Papilio* and *Heliconius* (Sweeney et al., 2003) and, as demonstrated in the former, polarized light is not even perceived as such but just modulates luminance perception. The water-surface detectors of water-dependent insects and the ventral polarization sense of *Drosophila* may well be non-imaging and designed for wide-field stimuli. Why does exact e-vector analysis have such a low importance in object-based polarization vision?

Under terrestrial conditions, the use of e-vector orientation for detecting or identifying objects is unfavorable for two reasons. First, terrestrial background can contain strong polarization noise. For instance, the light reflected from shiny leaves of vegetation can be polarized, thereby masking polarized objects of interest (see fig. 19.1a in Marshall et al., 2014; fig. 8.12 in Wehner and Labhart, 2006). Second, the e-vector orientation of reflected light depends on both the orientation of the reflecting surfaces and the position of the light source. Therefore, for an organism equipped with e-vector perception, the appearance of one and the same object would change when viewed from different positions and/or under different illuminations. This is different to color vision, because the spectral properties of reflected light are less dependent on object orientation, as everyday experience tells us.

As an exception, the e-vector orientation of light reflected from water bodies (Fig. 1B) and mud flats is predictably horizontal. Strong activity of the horizontal analyzer channel combined with low activity of the vertical channel allows flying backswimmers to identify water surfaces reliably, and it provides fiddler crabs on mud flats with a visual background against which weakly polarized targets can easily be detected (How et al., 2015). In both cases, there is no need for precise e-vector analysis.

Underwater, there is little background polarization noise because the difference between the refraction indices of water and solid matter is comparatively small. This allowed cephalopods and mantis shrimp to develop their own, intrinsically produced polarization signals on their bodies for visual communication. It appears that both optical and chemical mechanisms are used to achieve this (Chiou et al., 2007; Chiou et al., 2012; Roberts et al., 2009). Although their dipolatic systems prohibit e-vector perception, the horizontal polarization of their somatic patterns (Chiou et al., 2007; Cronin et al., 2003; Marshall et al., 2014; Mäthger et al., 2009; Shashar et al., 1996) produces strong contrast signals on an unpolarized background (Fig. 1C). Apart from detecting the body patterns of conspecifics, horizontal/vertical dipolatic systems can reduce the mostly horizontally polarized haze in the water column (Fig. 1D), and they aid the detection of polarization-active, camouflaged transparent prey, or predators, which may be invisible by luminosity contrast alone (Johnsen et al., 2011; Schechner et al., 2003). Marshall et al. (2014) and How and Marshall (2014) suppose that, in contexts such as this, degree contrast is more reliable for object detection than e-vector contrast, as it is less dependent on the direction of illumination and object orientation.

### Celestial compass

The polarization compass is the only application of polarization vision in which directional information is crucial, by definition. The multidimensional DRAs of insects would, in principle, be suitable for e-vector perception. However, both electrophysiological and behavioral data from several insect species indicate that the celestial compass is not a monomodal system, which relies on skylight polarization alone, but also exploits the spectral and intensity gradients in the sky as well as the solar azimuth. While this multimodality excludes e-vector perception, it increases the robustness of the compass. The existence of polarization-sensitive neurons with time-of-day dependent e-vector tuning axes in locusts and monarch butterflies suggests that the output signal provided by the compass indicates a geographical azimuth (Heinze and Reppert, 2011; Merlin et al., 2012; Pfeiffer and Homberg, 2007), to which the celestial polarization pattern makes a crucial but not the only contribution.

Under laboratory conditions, directional information can be restricted to e-vector orientation, excluding all other celestial cues. This can also occur in the field, for instance because of a break in cloud cover or in a dense tree canopy. Will insects, in which e-vector analysis has been demonstrated to operate in the simultaneous mode (crickets: T.L., unpublished observations; bees: Sakura et al. (2012); locusts: Mappes and Homberg, 2004), perceive e-vector orientation as a separate modality of light in this situation? Probably not, because the purpose of the system will hardly change with the stimulus conditions, and its output will always indicate a direction.

### The benefits of dipolatic systems

In spite of their limited e-vector analyzing capacities, orthogonal 2D systems provide highly useful sensory modules. Receiving opponent input from the two analyzer channels, the polop neurons act as differential polarization sensors, the benefits of which can be summarized as follows. (1) Polop neurons sort e-vector orientations instantaneously into one of two perceptual categories, e.g. ‘vertical’ or ‘horizontal’, according to the polarity of the output signal. (2) Polop neurons effectively enhance the response to a polarized stimulus. (3) Comparable to differential amplifiers, they abolish the common luminance component of the two photoreceptor responses, i.e. the polop output becomes luminance independent. (4) Although a dipolat cannot disentangle e-vector and degree, whenever the polarization distance from the background reaches a threshold, polarization-active objects producing no luminance contrast may be detected by their polarization properties alone.

The practically universal presence of 2D input stages is suggestive of, but does not prove, the existence of antagonistic polarization analysis by polop neurons. Do polop neurons actually exist? The common presence of polarization-opponent neurons in the insect brain (reviewed by Heinze, 2014a) and evidence from a crayfish study (Glantz, 2001) indicate that the benefits of polarization antagonism are indeed exploited. On the behavioral level, backswimmers show a diving response to a ventral, horizontally polarized UV stimulus but remain unresponsive to vertically polarized or unpolarized light, which can only be explained by polarization-opponent processing (Schwind, 1984). For imaging polarization vision, polop neurons receiving input from individual ommatidia (elementary polarization detectors) are essential, but to my knowledge, no one has been able to identify them so far.

In non-imaging polarized skylight navigation, elementary polarization detectors are not necessarily required. Thus, cricket POL1 neurons receive convergent input from a large number of DRA ommatidia (Labhart et al., 2001). But note that the response properties of certain locust neurons (e.g. LoTu1) are incompatible with the polop mechanism, but must be explained by a dynamic process involving inhibitory receptor outputs alone (Pfeiffer et al., 2011). And, in *Drosophila*, reciprocal inhibition between the polarization-sensitive UV receptors R7 and R8 of the DRA was recently observed (Weir et al., 2016). This enhances PS of both receptors and may even replace polop neuron function under certain conditions (Weir et al., 2016).

The output signals provided by polop neurons are exploited in two ways: (1) in object-based polarization vision and (2) in the celestial compass, as discussed below.

#### The role of polop neurons in object-based polarization vision

In object-based, imaging polarization vision, each polop unit represents a pixel in a pure polarization image (Figs 4B, 5A). The pixels contain no precise information on e-vector orientation, they just indicate an e-vector range (Fig. 3D, H versus V). But ‘saturated’ pixels (strong polop signals) suggest good alignment of the stimulus e-vector with the e-vector tuning axis of one of the two analyzers and/or high degree. ‘Unsaturated’ pixels (weak polop signals) suggest poor alignment and/or weak degree. Underwater and on mud flats, the imaging polarization vision systems of cephalopods and crabs can reveal polarization-active objects, which may be hidden on a luminance basis, by e-vector and/or degree contrast against the background. In addition, polop neurons effectively reduce the predominantly horizontally polarized background haze in the water column, improving general visibility (Fig. 1D).

#### The role of polop neurons in the celestial compass

In polarization vision systems used for navigation purposes, where rough categorizing of e-vector orientations does not suffice and where gaining exact directional information is crucial, the dipolatic ommatidia of the DRA represent just the first level of analysis. Polarization antagonism provides enhanced, differential input signals to the actual e-vector analyzing system, which must be at least three-dimensional. The insect DRA represents such a multidimensional system, as the orientation of the ommatidia exhibits a fan-like gradient along the DRA, providing a wide spectrum of differently oriented analyzer pairs. Interestingly, in crickets, the retinal multidimensionality is reduced to 3D in the POL1 neuron system (Fig. 5B, lower row).

### E-vector perception versus true polarization vision

As explained above, a 2D system already suffices for true polarization vision, such that e-vector category may be perceived as a separate modality of light. However, to identify specific e-vector orientations (e-vector perception), a ≥3D system is required. While true polarization vision is easily defined, its formal confirmation calls for elaborate behavioral tests (Wehner, 2001); thus, just a handful of proven cases are known. Luminance and spectral independence has been demonstrated for water-surface detection by backswimmers (Schwind, 1983, 1984), and luminance insensitivity was found for the object-based polarization vision of mantis shrimp (Marshall et al., 1999). In cuttlefish, luminance and polarization information seem to be processed by separate paths (Cartron et al., 2013). But most behavioral observations can be explained by the mere presence of polarization-sensitive photoreceptors without opponent signal interactions (Fig. 4C), at least qualitatively (crabs: How et al., 2012, 2014; cephalopods: Moody and Parriss, 1961; Pignatelli et al., 2011; Shashar and Cronin, 1996; Shashar et al., 1996; Temple et al., 2012). This is because even 1D polarization vision with a retina containing just one polarization-sensitive e-vector type of photoreceptor (e.g. horizontal) can convert pure polarization-contrast images to luminance-contrast images, in the same way as looking through a single, stationary polarizing filter (with, for example a horizontal polarizing axis) can make pure polarization contrasts visible as brightness contrasts to human observers (e.g. movie 1 in Temple et al., 2012; fig. 1 in Shashar and Cronin, 1996). Could a 1D system (or two independent 1D systems) also explain the observed low e-vector discrimination thresholds of 1 to 3 deg (How et al., 2012; Temple et al., 2012)? A quick, practical test involving visual inspection of two backlit polarizers with a 5 deg e-vector difference viewed through a polarizer shows a just-noticeable brightness difference at optimal analyzer orientation. Considering the comparatively much weaker analyzing power of polarization-sensitive photoreceptors, signal processing without the contrast-enhancing benefits of opponent processing seems questionable. The general occurrence of 2D input stages suggests that true polarization vision may be quite frequent in object-based polarization vision. However, it cannot be excluded that polarization and luminance images are combined at some level in the brain (How et al., 2014), confounding true polarization vision again.

## Conclusions

In both object-oriented polarization vision and water-surface detection, the orthogonally dipolatic ommatidia show a constant orientation in the retina such that the microvilli are directed horizontally or vertically (Fig. 5A). This arrangement does not allow subtle e-vector analysis, but dipolatic animals must perceive light polarization in a way analogous to how dichromats perceive colors. Although dipolats confuse e-vector and degree, polarized stimuli on either unpolarized or differently polarized backgrounds will, in many cases, produce detectable contrast signals. Apparently, object-based polarization vision is designed for detecting polarization contrasts, based on both e-vector and/or degree, rather than for absolute e-vector analysis. While the 2D structure of the ommatidia suggests true polarization vision, it remains unclear whether the polarization image remains separate from the brightness image or merges with it at some stage.

Although the ommatidia of the insect DRA, which serve as detectors for polarized skylight, are also dipolatic, they are oriented in a fan-like, multi-dimensional array (Fig. 5B) and are used to condition the incoming receptor signals. The DRAs of some insects would allow e-vector analysis, in principle, but these are non-imaging systems. In addition, the polarization-sensitive DRA represents just one of several input paths to a multimodal celestial compass system. Apparently, the function of the DRA is not to experience the celestial polarization pattern in detail, but to provide the compass with useful directional information.

In conclusion, to my knowledge, so far there exists no evidence that any invertebrate organism can perceive specific e-vector orientations of plane-polarized light. It seems that invertebrate organisms take no interest in the polarization details of visual stimuli, but they profit from PS by more practical benefits such as improved object detection and visual communication for cephalopods and some crustaceans, compass readings for traveling insects or the alert ‘water below!’ for water-seeking, flying bugs.

## References

[JEB139899C1] Alkaladi A., How M. J. and Zeil J. (2013). Systematic variations in microvilli banding patterns along fiddler crab rhabdoms. *J. Comp. Physiol. A* 199, 99-113. 10.1007/s00359-012-0771-9 23108879

[JEB139899C3] Bernard G. D. and Wehner R. (1977). Functional similarities between polarization vision and color vision. *Vision Res.* 17, 1019-1028. 10.1016/0042-6989(77)90005-0 595410

[JEB139899C4] Blum M. and Labhart T. (2000). Photoreceptor visual fields, ommatidial array, and receptor axon projections in the polarisation-sensitive dorsal rim area of the cricket compound eye. *J. Comp. Physiol. A* 186, 119-128. 10.1007/s003590050012 10707310

[JEB139899C5] Brunner D. and Labhart T. (1987). Behavioural evidence for polarization vision in crickets. *Physiol. Entomol.* 12, 1-10. 10.1111/j.1365-3032.1987.tb00718.x

[JEB139899C6] Cartron L., Dickel L., Shashar N. and Darmaillacq A.-S. (2013). Maturation of polarization and luminance contrast sensitivities in cuttlefish (*Sepia officinalis*). *J. Exp. Biol.* 216, 2039-2045. 10.1242/jeb.080390 23430993

[JEB139899C7] Chiou T.-H., Mäthger L. M., Hanlon R. T. and Cronin T. W. (2007). Spectral and spatial properties of polarized light reflections from the arms of squid (*Loligo pealeii*) and cuttlefish (*Sepia officinalis* L.). *J. Exp. Biol.* 210, 3624-3635. 10.1242/jeb.006932 17921164

[JEB139899C8] Chiou T.-H., Kleinlogel S., Cronin T. W., Caldwell R., Loeffler B., Siddiqi A., Goldizen A. and Marshall N. J. (2008). Circular polarization vision in a stomatopod crustacean. *Curr. Biol.* 18, 429-434. 10.1016/j.cub.2008.02.066 18356053

[JEB139899C9] Chiou T.-H., Place A. R., Caldwell R. L., Marshall N. J. and Cronin T. W. (2012). A novel function for a carotenoid: astaxanthin used as a polarizer for visual signalling in a mantis shrimp. *J. Exp. Biol.* 215, 584-589. 10.1242/jeb.066019 22279065

[JEB139899C10] Cronin T. W. and Marshall J. (2011). Patterns and properties of polarized light in air and water. *Philos. Trans. R. Soc. B Biol. Sci.* 366, 619-626. 10.1098/rstb.2010.0201 PMC3049010 21282165

[JEB139899C11] Cronin T. W., Shashar N., Caldwell R. L., Marshall N. J., Cheroske A. G. and Chiou T.-H. (2003). Polarization vision and its role in biological signaling. *Integr. Comp. Biol.* 43, 549-558. 10.1093/icb/43.4.549 21680463

[JEB139899C12] Dacke M. (2014). Polarized light orientation in ball-rolling dung beetles. In *Polarized Light and Polarization Vision in Animal Sciences* (ed. Horvath G.), pp. 27-39. Berlin; Heidelberg: Springer.

[JEB139899C13] Dacke M., Nilsson D.-E., Warrant E. J., Blest A. D., Land M. F. and O'Carroll D. C. (1999). Built-in polarizers form part of a compass organ in spiders. *Nature* 401, 470-473. 10.1038/46773

[JEB139899C14] Dacke M., Doan T. A. and O'Carroll D. C. (2001). Polarized light detection in spiders. *J. Exp. Biol.* 204, 2481-2490. 11511663 10.1242/jeb.204.14.2481

[JEB139899C15] Dacke M., El Jundi B., Smolka J., Byrne M. and Baird E. (2014). The role of the sun in the celestial compass of dung beetles. *Philos. Trans. R. Soc. B Biol. Sci.* 369, 20130036 10.1098/rstb.2013.0036 PMC3886324 24395963

[JEB139899C16] Daly I. M., How M. J., Partridge J. C., Temple S. E., Marshall N. J., Cronin T. W. and Roberts N. W. (2016). Dynamic polarization vision in mantis shrimps. *Nat. Commun.* 7, 12140 10.1038/ncomms12140 27401817 PMC4945877

[JEB139899C17] Dyer F. C. and Gould J. L. (1983). Honey bee navigation: the honey bee's ability to find its way on a hierarchy of sophisticated orientation mechanisms. *Am. Sci.* 71, 587-597.

[JEB139899C18] el Jundi B., Pfeiffer K., Heinze S. and Homberg U. (2014a). Integration of polarization and chromatic cues in the insect sky compass. *J. Comp. Physiol. A* 200, 575-589. 10.1007/s00359-014-0890-6 24589854

[JEB139899C19] el Jundi B., Smolka J., Baird E., Byrne M. and Dacke M. (2014b). Diurnal dung beetles use the intensity gradient and the polarization pattern of the sky for orientation. *J. Exp. Biol.* 217, 2422-2429. 10.1242/jeb.101154 24737763

[JEB139899C20] el Jundi B., Warrant E. J., Byrne E. J., Khaldy L. Baird E., Smolka J. and Dacke M. (2015). Neural coding underlying the cue preference for celestial orientation. *Proc. Natl. Acad. Sci. USA* 112, 11395-11400. 10.1073/pnas.1501272112 26305929 PMC4568659

[JEB139899C21] Fischer S., Meyer-Rochow V. B. and Müller C. H. G. (2014). Compound eye miniaturization in Lepidoptera: a comparative morphological analysis. *Acta Zool.* 95, 438-464. 10.1111/azo.12041

[JEB139899C22] Foster J. J., Sharkey C. R., Gawrowska A. V. A., Roberts N. W., Whitney H. M. and Partridge J. (2014). Bumblebees learn polarization patterns. *Curr. Biol.* 24, 1415-1420. 10.1016/j.cub.2014.05.007 24909321 PMC4062934

[JEB139899C23] Geiser F. X. (1985). Elektrophysiologische Charakterisierung der Ocellen von *Apis mellifera* und *Cataglyphis bicolor*. *PhD thesis*, University of Zurich.

[JEB139899C24] Geiser F. X. and Labhart T. (1982). Elektrophysiologische Untersuchungen an der Ocellen-Retina der Honigbiene (*Apis mellifera*). *Verh. Dtsch. Zool. Ges.* 1982, 307.

[JEB139899C25] Glantz R. M. (2001). Polarization analysis in the crayfish visual system. *J. Exp. Biol.* 204, 2383-2390. 11511653

[JEB139899C26] Glantz R. M. (2007). The distribution of polarization sensitivity in the crayfish retina. *J. Comp. Physiol. A* 193, 893-901. 10.1007/s00359-007-0242-x 17598114

[JEB139899C27] Glantz R. M. (2008). Polarization vision in crayfish motion detectors. *J. Comp. Physiol. A* 194, 565-575. 10.1007/s00359-008-0331-5 18386016

[JEB139899C28] Goldsmith T. H. and Wehner R. (1977). Restrictions on rotational and translational diffusion of pigment in the membranes of a rhabdomeric photoreceptor. *J. Gen. Physiol.* 70, 453-490. 10.1085/jgp.70.4.453 410904 PMC2228504

[JEB139899C29] Hardie R. C. (1984). Properties of photoreceptors R7 and R8 in dorsal marginal ommatidia in the compound eyes of *Musca* and *Calliphora*. *J. Comp. Physiol. A* 154, 157-165. 10.1007/BF00604981

[JEB139899C30] Hardie R. C. (1985). Functional organization of the fly retina. In *Progress in Sensory Physiology* (ed. Hardie R. C.), pp. 3-79. New York: Springer.

[JEB139899C31] Heinze S. (2014a). Polarized-light processing in insect brains: recent insights from the desert locust, the monarch butterfly, the cricket, and the fruit fly. In *Polarized Light and Polarization Vision in Animal Sciences* (ed. Horvath G.), pp. 61-111. Berlin; Heidelberg: Springer.

[JEB139899C32] Heinze S. (2014b). Polarization vision. In *Encyclopedia of Computational Neuroscience* (ed. Jäger D. and Jung R.), pp. 1-30. New York: Springer.

[JEB139899C33] Heinze S. and Reppert S. M. (2011). Sun compass integration of skylight cues in migratory monarch butterflies. *Neuron* 69, 345-358. 10.1016/j.neuron.2010.12.025 21262471

[JEB139899C34] Henze M. J. and Labhart T. (2007). Haze, clouds and limited sky visibility: polarotactic orientation of crickets under difficult stimulus conditions. *J. Exp. Biol.* 210, 3266-3276. 10.1242/jeb.007831 17766304

[JEB139899C35] Henze M. J., Dannenhauer K., Kohler M., Labhart T. and Gesemann M. (2012). Opsin evolution and expression in arthropod compound eyes and ocelli: insights from the cricket *Gryllus bimaculatus*. *BMC Evol. Biol.* 12, 163 10.1186/1471-2148-12-163 22935102 PMC3502269

[JEB139899C36] Horvath G. (2014). *Polarized Light and Polarization Vision in Animal Sciences*. Berlin; Heidelberg; New York: Springer.

[JEB139899C37] Horvath G. and Csabai Z. (2014). Polarization vision of aquatic insects. In *Polarized Light and Polarization Vision in Animal Sciences* (ed. Horvath G.), pp. 113-146. Berlin; Heidelberg: Springer.

[JEB139899C38] Horvath G. and Varju D. (2004). *Polarized Light in Animal Vision. Polarization Patterns in Nature*. Berlin; Heidelberg; New York: Springer.

[JEB139899C39] How M. J. and Marshall N. J. (2014). Polarization distance: a framework for modelling object detection by polarization vision systems. *Proc. R. Soc. B Biol. Sci.* 281, 20131632 10.1098/rspb.2013.1632 PMC3871304 24352940

[JEB139899C40] How M. J., Pignatelli V., Temple S. E., Marshall N. J. and Hemmi J. M. (2012). High e-vector acuity in the polarisation vision system of the fiddler crab *Uca vomeris*. *J. Exp. Biol.* 215, 2128-2134. 10.1242/jeb.068544 22623201

[JEB139899C41] How M. J., Christy J., Roberts N. W. and Marshall N. J. (2014). Null point of discrimination in crustacean polarisation vision. *J. Exp. Biol.* 217, 2462-2467. 10.1242/jeb.103457 24737768

[JEB139899C43] How M. J., Christy J. H., Temple S. E., Hemmi J. M., Marshall N. J. and Roberts N. W. (2015). Target detection is enhanced by polarization vision in a fiddler crab. *Curr. Biol.* 25, 3069-3073. 10.1016/j.cub.2015.09.073 26585278

[JEB139899C44] Israelachvili J. N. and Wilson M. (1976). Absorption characteristics of oriented photopigments in microvilli. *Biol. Cybern.* 21, 9-15. 10.1007/BF00326667 1244867

[JEB139899C45] Johnsen S., Marshall N. J. and Widder E. A. (2011). Polarization sensitivity as a contrast enhancer in pelagic predators: lessons from in situ polarization imaging of transparent zooplankton. *Philos. Trans. R. Soc. B Biol. Sci.* 366, 655-670. 10.1098/rstb.2010.0193 PMC3049004 21282169

[JEB139899C46] Kelber A., Thunell C. and Arikawa K. (2001). Polarization-dependent color vision in *Papilio* butterflies. *J. Exp. Biol.* 204, 2469-2480. 11511662 10.1242/jeb.204.14.2469

[JEB139899C47] Kinoshita M., Yamazato K. and Arikawa K. (2011). Polarization-based brightness discrimination in the foraging butterfly, *Papilio xuthus*. *Philos. Trans. R. Soc. B Biol. Sci.* 366, 688-696. 10.1098/rstb.2010.0200 PMC3049009 21282172

[JEB139899C48] Kirschfeld K. (1969). Absorption properties of photopigments in single rods and rhabdomeres. In *Processing of Optical Data by Organisms and Machines* (ed. Reichardt W.), pp. 116-136. New York: Academic Press.

[JEB139899C49] Kirschfeld K. (1972). Notizen: Die notwendige Anzahl von Rezeptoren zur Bestimmung der Richtung des elektrischen Vektors linear polarisierten Lichtes. *Z. Naturforsch.* 27, 578-579. 10.1515/znb-1972-0524 4403414

[JEB139899C50] Kleinlogel S. and Marshall N. J. (2005). Photoreceptor projection and termination pattern in the lamina of gonodactyloid stomatopods (mantis shrimp). *Cell Tissue Res.* 321, 273-284. 10.1007/s00441-005-1118-4 15947970

[JEB139899C51] Kleinlogel S. and Marshall N. J. (2006). Electrophysiological evidence for linear polarization sensitivity in the compound eyes of the stomatopod crustacean *Gonodactylus chiragra*. *J. Exp. Biol.* 209, 4262-4272. 10.1242/jeb.02499 17050841

[JEB139899C53] Labhart T. (1980). Specialized photoreceptors at the dorsal rim of the honeybee's compound eye: polarizational and angular sensitivity. *J. Comp. Physiol. A* 141, 19-30. 10.1007/BF00611874

[JEB139899C54] Labhart T. (1986). The electrophysiology of photoreceptors in different eye regions of the desert ant, *Cataglyphis bicolor*. *J. Comp. Physiol. A* 158, 1-7. 10.1007/BF00614514

[JEB139899C55] Labhart T. (1988). Polarization-opponent interneurons in the insect visual system. *Nature* 331, 435-437. 10.1038/331435a0

[JEB139899C56] Labhart T. and Meyer E. P. (1999). Detectors for polarized skylight in insects: a survey of ommatidial specializations in the dorsal rim area of the compound eye. *Microsc. Res. Tech.* 47, 368-379. 10.1002/(SICI)1097-0029(19991215)47:6<368::AID-JEMT2>3.0.CO;2-Q 10607378

[JEB139899C57] Labhart T. and Meyer E. P. (2002). Neural mechanisms in insect navigation: polarization compass and odometer. *Curr. Opin. Neurobiol.* 12, 707-714. 10.1016/S0959-4388(02)00384-7 12490263

[JEB139899C58] Labhart T. and Petzold J. (1993). Processing of polarized light information in the visual system of crickets. In *Sensory Systems of Arthropods* (ed. Wiese K., Gribakin F. G., Popov A. V. and Renninger G.), pp. 158-168. Basel; Boston; Berlin: Birkhäuser.

[JEB139899C59] Labhart T., Meyer E. P. and Schenker L. (1992). Specialized ommatidia for polarization vision in the compound eye of cockchafers, *Melolontha melolontha* (Coleoptera, Scarabaeidae). *Cell Tissue Res.* 268, 419-429. 10.1007/BF00319148 1628299

[JEB139899C60] Labhart T., Petzold J. and Helbling H. (2001). Spatial integration of polarization-sensitive interneurones of crickets: a survey of evidence, mechanisms and benefits. *J. Exp. Biol.* 2004, 2423-2430. 10.1242/jeb.204.14.2423 11511657

[JEB139899C61] Labhart T., Baumann F. and Bernard G. D. (2009). Specialized ommatidia of the polarization-sensitive dorsal rim area in the eye of monarch butterflies have non-functional reflecting tapeta. *Cell Tissue Res.* 338, 391-400. 10.1007/s00441-009-0886-7 19876649 PMC2779342

[JEB139899C62] Lambrinos D., Kobayashi H., Pfeifer R., Maris M., Labhart T. and Wehner R. (1997). An autonomous agent navigating with a polarized light compass. *Adapt. Behav.* 6, 131-161. 10.1177/105971239700600104

[JEB139899C63] Lambrinos D., Möller R., Labhart T., Pfeifer R. and Wehner R. (2000). A mobile robot employing insect strategies for navigation. *Rob. Auton. Syst.* 30, 39-64. 10.1016/S0921-8890(99)00064-0

[JEB139899C64] Land M. F., Marshall J. N., Brownless D. and Cronin T. W. (1990). The eye-movements of the mantis shrimp *Odontodactylus scyllarus* (Crustacea: Stomatopoda). *J. Comp. Physiol. A* 167, 155-166. 10.1007/BF00188107

[JEB139899C65] Lau D. (1976). Reaktionen von Honigbienen auf Polarisationsmuster an der Futterquelle. *Zool. Garten. Neue Folge Jena* 46, 34-38.

[JEB139899C66] Lerner A. (2014). Polarization as a guiding cue for oviposition of non-biting midges and mosquitoes. In *Polarized Light and Polarization Vision in Animal Sciences* (ed. Horvath G.), pp. 517-523. Berlin; Heidelberg: Springer.

[JEB139899C67] Mappes M. and Homberg U. (2004). Behavioral analysis of polarization vision in tethered flying locusts. *J. Comp. Physiol. A* 190, 61-68. 10.1007/s00359-003-0473-4 14648100

[JEB139899C68] Marshall N. J. (1988). A unique colour and polarization vision system in mantis shrimps. *Nature* 333, 557-560. 10.1038/333557a0 3374602

[JEB139899C69] Marshall N. J. and Cronin T. W. (2014). Polarization vision of crustaceans. In *Polarized Light and Polarization Vision in Animal Sciences* (ed Horvath G.), pp. 171-216. Berlin; Heidelberg: Springer.

[JEB139899C70] Marshall N. J., Land M. F., King C. A. and Cronin T. W. (1991). The compound eyes of mantis shrimps (Crustacea, Hoplocarida, Stomatopoda). I. Compound eye structure: the detection of polarized light. *Philos. Trans. R. Soc. B Biol. Sci.* 334, 33-56. 10.1098/rstb.1991.0096

[JEB139899C71] Marshall N. J., Cronin T. W., Shashar N. and Land M. (1999). Behavioural evidence for polarisation vision in stomatopods reveals a potential channel for communication. *Curr. Biol.* 9, 755-758. 10.1016/S0960-9822(99)80336-4 10421580

[JEB139899C72] Marshall N. J., Cronin T. W. and Wehling M. F. (2011). New directions in biological research on polarized light. *Philos. Trans. R. Soc. B Biol. Sci.* 366, 611-782. 10.1098/rstb.2010.0332 PMC3049018 21282163

[JEB139899C73] Marshall N. J., Roberts N. Cronin T. W. (2014). Polarization signals. In *Polarized Light and Polarization Vision in Animal Sciences* (ed. Horvath G.), pp. 407-441. Berlin; Heidelberg: Springer.

[JEB139899C74] Mäthger L. M., Shashar N. and Hanlon R. T. (2009). Do cephalopods communicate using polarized light reflections from their skin? *J. Exp. Biol.* 212, 2133-2140. 10.1242/jeb.020800 19561202

[JEB139899C75] Merlin C., Heinze S. and Reppert S. (2012). Unraveling navigational strategies in migratory insects. *Curr. Opin.* 22, 353-361. 10.1016/j.conb.2011.11.009 PMC3306460 22154565

[JEB139899C76] Moody M. F. and Parriss J. R. (1961). The discrimination of polarized light by *Octopus*: a behavioural and morphological study. *Z. Vergl. Physiol.* 44, 268-291. 10.1007/BF00298356

[JEB139899C77] Mote M. I. and Wehner R. (1980). Functional characteristics of photoreceptors in the compound eye and ocellus of the desert ant, *Cataglyphis bicolor*. *J. Comp. Physiol. A* 137, 63-71. 10.1007/BF00656918

[JEB139899C78] Mueller K. P. and Labhart T. (2010). Polarizing optics in a spider eye. *J. Comp. Physiol. A* 196, 335-348. 10.1007/s00359-010-0516-6 20229246

[JEB139899C79] Neitz J., Geist T. and Jacobs G. H. (1989). Color vision in the dog. *Vis. Neurosci.* 3, 119-125. 10.1017/S0952523800004430 2487095

[JEB139899C80] Neitz J., Carroll J. and Neitz M. (2001). Color vision: almost reason enough for having eyes. *Opt. Photonic News* 12, 26-33. 10.1364/OPN.12.1.000026

[JEB139899C81] Nilsson D.-E. and Warrant E. J. (1999). Visual discrimination: seeing the third quality of light. *Curr. Biol.* 9, R535-R537. 10.1016/S0960-9822(99)80330-3 10421572

[JEB139899C82] Nilsson D.-E., Labhart T. and Meyer E. P. (1987). Photoreceptor design and optical properties affecting polarization sensitivity in ants and crickets. *J. Comp. Physiol. A* 161, 645-658. 10.1007/BF00605006

[JEB139899C83] Pfeiffer K. and Homberg U. (2007). Coding of azimuthal directions via time-compensated combination of celestial compass cues. *Curr. Biol.* 17, 960-965. 10.1016/j.cub.2007.04.059 17524646

[JEB139899C84] Pfeiffer K., Negrello M. and Homberg U. (2011). Conditional perception under stimulus ambiguity: polarization- and azimuth-sensitive neurons in the locust brain are inhibited by low degrees of polarization. *J. Neurophysiol.* 105, 28-35. 10.1152/jn.00480.2010 20962068

[JEB139899C85] Pignatelli V., Temple S. E., Chiou T.-H. Roberts N. W., Collin S. P. and Marshall N. J. (2011). Behavioural relevance of polarization sensitivity as a target detection mechanism in cephalopods and fishes. *Philos. Trans. R. Soc. B Biol. Sci.* 366, 734-741. 10.1098/rstb.2010.0204 PMC3049012 21282177

[JEB139899C86] Reppert S. M., Zhu H. and White R. H. (2004). Polarized light helps monarch butterflies navigate. *Curr. Biol.* 14, 155-158. 10.1016/j.cub.2003.12.034 14738739

[JEB139899C87] Ribi W. A. (1980). New aspects of polarized light detection in the bee in view of non-twisting rhabdomeric structures. *J. Comp. Physiol.* 137, 281-285. 10.1007/BF00657124

[JEB139899C88] Roberts N. W., Chiou T.-H. Marshall N. J. and Cronin T. W. (2009). A biological quarter-wave retarder with excellent achromaticity in the visible wavelength region. *Nat. Photonics* 3, 641-644. 10.1038/nphoton.2009.189

[JEB139899C89] Roberts N. W., Porter M. L. and Cronin T. W. (2011). The molecular basis of mechanisms underlying polarization vision. *Philos. Trans. R. Soc. B Biol. Sci.* 366, 627-637. 10.1098/rstb.2010.0206 PMC3049014 21282166

[JEB139899C90] Rossel S. and Wehner R. (1984). Celestial orientation in bees: the use of spectral cues. *J. Comp. Physiol. A* 155, 605-613. 10.1007/BF00610846

[JEB139899C91] Saidel W. M., Lettvin J. Y. and MacNichol E. F. Jr. (1983). Processing of polarized light by squid photoreceptors. *Nature* 304, 534-536. 10.1038/304534a0 6877374

[JEB139899C92] Sakura M., Lambrinos D. and Labhart T. (2008). Polarized skylight navigation in insects: model and electrophysiology of e-vector coding by neurons in the central complex. *J. Neurophysiol.* 99, 667-682. 10.1152/jn.00784.2007 18057112

[JEB139899C93] Sakura M., Okada R. and Aonuma H. (2012). Evidence for instantaneous e-vector detection in the honeybee using an associative learning paradigm. *Proc. R. Soc. B Biol. Sci*. 279, 535-542. 10.1098/rspb.2011.0929 PMC3234551 21733901

[JEB139899C94] Schechner Y. Y. and Karpel N. (2004). Clear underwater vision. *Proc. Comp. Vis. Pattern Recog.* 1, 536-543. 10.1109/cvpr.2004.1315078

[JEB139899C95] Schechner Y. Y., Narasimhan S. G. and Nayar S. K. (2003). Polarization-based vision through haze. *Appl. Opt.* 42, 511-525. 10.1364/AO.42.000511 12570274

[JEB139899C96] Schmeling F., Tegtmeier J., Kinoshita M. and Homberg U. (2015). Photoreceptor projections and receptive fields in the dorsal rim area and main retina of the locust eye. *J. Comp. Physiol. A* 201, 427-440. 10.1007/s00359-015-0990-y 25715758

[JEB139899C97] Schwind R. (1983). Zonation of the optical environment and zonation in the rhabdom structure within the eye of the backswimmer, *Notonecta glauca*. *Cell Tissue Res.* 232, 53-63. 10.1007/BF00222373 6883440

[JEB139899C98] Schwind R. (1984). Evidence for true polarization vision based on a two-channel analyzer system in the eye of the water bug, *Notonecta glauca*. *J. Comp. Physiol. A* 154, 53-57. 10.1007/BF00605390

[JEB139899C99] Schwind R. (1991). Polarization vision in water insects and insects living on a moist substrate. *J. Comp. Physiol. A* 169, 531-540. 10.1007/BF00193544

[JEB139899C100] Shashar N. and Cronin T. W. (1996). Polarization contrast vision in *Octopus*. *J. Exp. Biol.* 199, 999-1004. 8788092 10.1242/jeb.199.4.999

[JEB139899C101] Shashar N., Rutledge P. S. and Cronin T. W. (1996). Polarization vision in cuttlefish – a concealed communication channel? *J. Exp. Biol.* 199, 2077-2084. 9319987 10.1242/jeb.199.9.2077

[JEB139899C102] Shashar N., Hanlon R. T. and Petz A. M. (1998). Polarization vision helps detect transparent prey. *Nature* 393, 222-223. 10.1038/303809607759

[JEB139899C103] Stalleicken J., Labhart T. and Mouritsen H. (2006). Physiological characterization of the compound eye in monarch butterflies with focus on the dorsal rim area. *J. Comp. Physiol. A* 192, 321-331. 10.1007/s00359-005-0073-6 16317560

[JEB139899C104] Sweeney A., Jiggins C. and Johnsen S. (2003). Insect communication: polarized light as a butterfly mating signal. *Nature* 423, 31-32. 10.1038/423031a 12721616

[JEB139899C105] Talbot C. M. and Marshall J. N. (2011). The retinal topography of three species of coleoid cephalopod: significance for perception of polarized light. *Philos. Trans. R. Soc. B Biol. Sci.* 366, 724-733. 10.1098/rstb.2010.0254 PMC3049017 21282176

[JEB139899C106] Taylor G., Ribi W., Bech M., Bodey A. J., Rau C., Steuwer A., Warrant E. J. and Baird E. (2016). The dual function of orchid bee ocelli as revealed by X-ray microtomography. *Curr. Biol.* 26, 1319-1324. 10.1016/j.cub.2016.03.038 27112298

[JEB139899C107] Temple S. E., Pignatelli V., Cook T., How M. J., Chiou T.-H., Roberts N. W. and Marshall N. J. (2012). High resolution polarization vision in a cuttlefish. *Curr. Biol.* 22, R121-R122. 10.1016/j.cub.2012.01.010 22361145

[JEB139899C108] Vienot F., Brettel H., Ott L., M'Arek A. B. and Mollon J. D. (1995). What do colour-blind people see? *Nature* 376, 127-128. 10.1038/376127a0 7603561

[JEB139899C109] Waterman T. H. and Horch K. W. (1966). Mechanism of polarized light perception. *Science* 154, 467-475. 10.1126/science.154.3748.467 5916942

[JEB139899C110] Wehner R. (1982). Himmelsnavigation bei Insekten. *Neujahrsbl. Naturforsch. Ges. Zürich* 5, 1-132.

[JEB139899C111] Wehner R. (1997). The ant's celestial compass system: spectral and polarizational channels. In *Orientation and Communication in Arthropods* (ed. Lehrer M.), pp. 145-185. Basel: Birkhäuser.

[JEB139899C112] Wehner R. (2001). Polarization vision: a uniform sensory capacity? *J. Exp. Biol.* 204, 2589-2596. 11511675 10.1242/jeb.204.14.2589

[JEB139899C113] Wehner R. (2014). Polarization vision: a discovery story. In *Polarized Light and Polarization Vision in Animal Sciences* (ed. Horvath G.), pp. 3-25. Berlin; Heidelberg: Springer.

[JEB139899C114] Wehner R. and Labhart T. (2006). Polarization vision. In *Invertebrate Vision* (ed. Warrant E.J. and Nilsson D.-E.), pp. 291-348. Cambridge: Cambridge University Press.

[JEB139899C115] Wehner R. and Müller M. (2006). The significance of direct sunlight and polarized skylight in the ant's celestial system of navigation. *Proc. Natl. Acad. Sci. USA* 103, 12575-12579. 10.1073/pnas.0604430103 16888039 PMC1567920

[JEB139899C116] Wehner R. and Strasser S. (1985). The POL area of the honey bee's eye: behavioural evidence. *Physiol. Entomol.* 10, 337-349. 10.1111/j.1365-3032.1985.tb00055.x

[JEB139899C117] Wehner R., Bernard G. D. and Geiger E. (1975). Twisted and non-twisted rhabdoms and their significance for polarization detection in the bee. *J. Comp. Physiol. A* 104, 225-245. 10.1007/BF01379050

[JEB139899C118] Weir P., Henze M., Bleul C., Baumann-Klausener F., Labhart T. and Dickinson M. (2016). Anatomical reconstruction and functional imaging reveal an ordered array of skylight polarization detectors in *Drosophila*. *J. Neurosci.* 36, 5397-5404. 10.1523/JNEUROSCI.0310-16.2016 27170135 PMC4863064

[JEB139899C119] Wernet M. F., Labhart T., Baumann F., Mazzoni E. O., Pichaud F. and Desplan C. (2003). Homothorax switches function of *Drosophila* photoreceptors from color to polarized light sensors. *Cell* 115, 267-279. 10.1016/S0092-8674(03)00848-1 14636555

[JEB139899C120] Wernet M. F., Velez M. M., Clark D. A., Baumann-Klausener F., Brown J. R., Klovstad M., Labhart T. and Clandinin T. R. (2012). Genetic dissection reveals two separate retinal substrates for polarization vision in *Drosophila*. *Curr. Biol.* 22, 12-20. 10.1016/j.cub.2011.11.028 22177904 PMC3258365

[JEB139899C121] Wildermuth H. (1998). Dragonflies recognize the water of rendezvous and oviposition sites by horizontally polarized light: a behavioural field test. *Naturwissenschaften* 85, 297-302. 10.1007/s001140050504

[JEB139899C122] Yamahama Y., Hironaka T. and Hariyama T. (2014). Regional differences in photoreceptor structures in the compound eye of the stinkbug, *Plautia crossota* stali. *Jpn. J. Appl. Entomol. Zool.* 58, 319-327. 10.1303/jjaez.2014.319

[JEB139899C123] Zeil J., Ribi W. A. and Narendra A. (2014). Polarization vision in ants, bees and wasps. In *Polarized Light and Polarization Vision in Animal Sciences* (ed. Horvath G.), pp. 41-60. Berlin; Heidelberg: Springer.

